# Neuropsychiatric Symptom Clusters and their Association with Function and Brain Structure

**DOI:** 10.1002/alz70856_103652

**Published:** 2025-12-24

**Authors:** Daniel Kapustin, Neda Rashidi‐Ranjbar, Wei Wang, Malcolm Binns, Paula M McLaughlin, Agessandro Abrahao, David A. Grimes, Anthony E Lang, Connie Marras, Mario Masellis, Joseph B Orange, Tarek K. Rajji, Angela C. Roberts, Gustavo Saposnik, Richard H. Swartz, David F. Tang‐Wai, Carmela Tartaglia, Angela Troyer, Corinne E. Fischer, Sanjeev Kumar

**Affiliations:** ^1^ University of Toronto, Toronto, ON, Canada; ^2^ Keenan Research Centre for Biomedical Science, Li Ka Shing Knowledge Institute, St. Michael's Hospital, Toronto, ON, Canada; ^3^ Centre for Addiction and Mental Health, Toronto, ON, Canada; ^4^ Rotman Research Institute, Baycrest Academy for Research and Education, Toronto, ON, Canada; ^5^ Nova Scotia Health, Halifax, NS, Canada; ^6^ Associate scientist, Evaluative Clinical Sciences, Hurvitz Brain Sciences Research Program, Sunnybrook Research Institute, Toronto, ON, Canada; ^7^ Neurology/Medicine, University of Ottawa, Ottawa, ON, Canada; ^8^ The Edmond J. Safra Program in Parkinson's Disease and Morton and Gloria Shulman Movement Disorders Clinic, Toronto, ON, Canada; ^9^ University Health Network, Toronto, ON, Canada; ^10^ Krembil Research Institute, University Health Network, Toronto, ON, Canada; ^11^ Sunnybrook Health Sciences Centre, Toronto, ON, Canada; ^12^ School of Communication, Northwestern University, Evanston, IL, USA; ^13^ Adult Neurodevelopment and Geriatric Psychiatry Division, CAMH, Toronto, ON, Canada; ^14^ Northwestern University, Evanston, IL, USA; ^15^ Western University, London, ON, Canada; ^16^ Li Ka Shing Knowledge Institute, Toronto, ON, Canada; ^17^ Sunnybrook Research Institute, Toronto, ON, Canada; ^18^ Department of Medicine, University of Toronto, Toronto, ON, Canada; ^19^ Krembil Brain Institute, University Health Network (UHN), Toronto, ON, Canada; ^20^ Tanz Centre for Research in Neurodegenerative Diseases, University of Toronto, Toronto, ON, Canada; ^21^ Baycrest Health Sciences Centre, Toronto, ON, Canada; ^22^ Department of Psychiatry, University of Toronto, Toronto, ON, Canada

## Abstract

**Background:**

Neuropsychiatric symptoms (NPS) constitute a major challenge for patients with Alzheimer's disease (AD). We explored NPS clusters in AD and longitudinally evaluated their association with function and structural neuroimaging markers.

**Method:**

Participants with AD (*N* = 111) were included from the Ontario Neurodegenerative Disease Research Initiative. NPS were assessed using the Neuropsychiatric Inventory Questionnaire (NPI‐Q). NPS clusters were identified through exploratory factor analysis at baseline. We evaluated 34 bilateral cortical regions of interest (ROIs) and 9 bilateral subcortical ROIs using volumetric information from MRI data evaluated annually over 3 years. We examined longitudinal associations between NPS clusters with basic (ADLs) and instrumental (iADLs) activities of daily living, as well as with subcortical and cortical volumes, using mixed linear regression models controlling for age, sex, MoCA, education level, and visit number.

**Result:**

Factor analysis identified four symptom clusters explaining 62% of the variance. These were labeled “behavioral” (disinhibition, irritability, motor disturbance, and agitation), “psychotic” (hallucinations, delusions, and euphoria), “neurovegetative” (apathy and appetite), and “affective” (depression, anxiety, nighttime behavior) clusters.

The “behavior” cluster was longitudinally associated with left middle temporal (β = ‐382.6, *p* = .006), right lingual (β = ‐456.0, *p* = .01), right nucleus accumbens (β = ‐5656.6, *p* = .004), and right thalamic (β = ‐700.7, *p* = .008) volumes. The “neurovegetative” cluster was longitudinally associated with left fusiform (β = ‐195.4, *p* = .03), left middle temporal (β = ‐282.3, *p* = .001), and right nucleus accumbens (β = ‐3176.7, *p* = .01). The “affective” cluster was associated with left rostral anterior cingulate (β = ‐1423.4, *p* = .0003), right entorhinal (β = ‐804.3, *p* = .03), right medial orbitofrontal (β = ‐830.6, *p* = .001), right pars opercularis (β = ‐911.6, *p* = .001), and left putamen (β = ‐729.5, *p* = .004) volumes.

Longitudinally, all clusters predicted iADL outcomes and clusters 1, 3, and 4 predicted ADL outcomes. Greater NPS burden, male sex, visit number, older age, and lower MoCA predicted worse function.

**Conclusion:**

NPS clusters in AD separate into behavioral, psychotic, neurovegetative, and affective dimensions. These clusters demonstrate unique associations with function and neuroimaging markers.

